# Correlation of clinical illness with viremia in Zika virus disease during an outbreak in Singapore

**DOI:** 10.1186/s12879-018-3211-9

**Published:** 2018-07-04

**Authors:** Deborah H. L. Ng, Hanley J. Ho, Angela Chow, Joshua Wong, Win Mar Kyaw, Adriana Tan, Po Ying Chia, Chiaw Yee Choy, Glorijoy Tan, Tsin Wen Yeo, Yee Sin Leo

**Affiliations:** 1 National Centre for Infectious Diseases, Moulmein Road, Singapore, 308433 Singapore; 2 Department of Clinical Epidemiology, Office of Clinical Epidemiology, Analytics, and Knowledge, Tan Tock Seng Hospital, 11 Jalan Tan Tock Seng, Singapore, 308433 Singapore; 3 Saw Swee Hock School of Public Health, National University of Singapore, 12 Science Drive 2, Singapore, 117549 Singapore; 4 Lee Kong Chian School of Medicine, Nanyang Technological University, 11 Mandalay Road, Singapore, 308232 Singapore

**Keywords:** Viremia, Zika, Outbreak, Singapore

## Abstract

**Background:**

The first autochthonous Zika virus (ZIKV) outbreak in Singapore was detected in August 2016. We report an analysis of the correlation of clinical illness with viremia and laboratory parameters in this Asian cohort.

**Methods:**

We conducted a prospective longitudinal cohort study of patients with a positive blood ZIKV polymerase chain reaction (PCR) result who were admitted to Tan Tock Seng Hospital, Singapore, for isolation and management.

**Results:**

We included 40 patients in our study. Rash was present in all patients, while 80% (32/40) had fever, 62.5% (25/40) myalgia, 60% (24/40) conjunctivitis and 38% (15/40) arthralgia. The median duration of viremia was 3.5 days (IQR: 3–5 days). Patients with viremia of **≥**4 days were more likely to have prolonged fever compared to those with viremia of less than 4 days (95% versus 63%, *p* = 0.01), but had no significant correlation with other clinical signs and symptoms, or laboratory investigations. However, 21 patients (53%) had hypokalemia despite the absence of gastrointestinal symptoms.

**Conclusion:**

Although fever correlated with duration of viremia, 30% of patients remained viremic despite defervescence. Laboratory abnormalities such as leukopenia or thrombocytopenia were not prominent in this cohort but about half the patients were noted to have hypokalemia.

## Background

Zika virus (ZIKV), a mosquito-borne flavivirus, gained international recognition following reports of microcephaly and Guillain-Barré syndrome (GBS) associated with the disease in South America, which had prompted the World Health Organisation (WHO) to declare it a Public Health Emergency of International Concern on February 1, 2016 [1]. However, the presence of ZIKV has been recognised in Southeast Asia for over half a century. Previous studies demonstrated serological evidence of ZIKV infections in Malaysia in 1969, and the first clinical infections were reported in Indonesia in 1977 [2].

On August 27, 2016, the first case of locally transmitted ZIKV disease in Singapore was identified. The patient was a 47-year-old female who had not travelled out of the country in the 2 weeks preceding her illness onset [3]. Active case finding by the Singapore Ministry of Health (MOH) revealed further laboratory-confirmed infections, signalling the presence of a large community outbreak of ZIKV. A total of 455 cases were reported up until 30 November 2016 [4]. During the initial outbreak containment phase, all suspected ZIKV infections were screened at Tan Tock Seng Hospital (TTSH), an acute tertiary care general hospital, for further management. Isolation at the Communicable Disease Center (CDC) was mandated by MOH for all cases with a positive blood ZIKV polymerase chain testing (PCR) result, with daily testing until results were negative before discharging them back to the community.

ZIKV generally causes a mild febrile illness, characterised by symptoms of rash, arthralgia, non-purulent conjunctivitis and/or low-grade fever [5]. Viremia in ZIKV disease has been reported to range from the day of onset of symptoms for up to 11 days from symptom onset [6]. However, little is known about the correlation between clinical symptoms and signs, as well as laboratory parameters, with viremia. Whole genome sequencing of the virus demonstrated that the ZIKV strain circulating in Singapore was genetically distinct from the strain imported from Brazil in May 2016 [4]. This study therefore aims to describe the clinical characteristics of ZIKV infection and its correlation to viremia and other laboratory investigations, as observed during the ZIKV outbreak in Singapore. As clinical symptoms such as fever are known to correlate with viremia for other arbovirus infections such as dengue, we hypothesised that a similar correlation might exist for ZIKV infection.

## Methods

We conducted a prospective longitudinal cohort study of patients with laboratory-confirmed ZIKV disease who were admitted to the CDC, TTSH from 26 August 2016 to 5 September 2016, when Singapore was in the containment phase of the ZIKV outbreak. TTSH is a 1500-bed teaching hospital, which also encompasses the CDC, the designated institution for centralised management of emerging infectious disease outbreaks in Singapore.

Active case finding for persons with suspected ZIKV disease was initiated by the MOH through public education and contact tracing. Adults living or working in the vicinity of the initial outbreak area with symptoms compatible with suspected ZIKV disease were advised to present directly to TTSH or were referred by primary care practitioners for further testing. The case definition for suspected ZIKV disease was any person with fever and maculopapular rash, plus one additional symptom of arthralgia, myalgia, headache or non-purulent conjunctivitis.

All suspect cases who fully or partially fulfilled the case definition had blood and urine samples tested for ZIKV ribonucleic acid (RNA) at Tan Tock Seng Hospital, using real-time reverse transcriptase polymerase chain reaction (rRT-PCR) methods as described by Lanciotti et al. [6] Blood and urine samples from the same specimens were also sent to the National Public Health Laboratory under the MOH, also for concurrent testing using rRT-PCR, for concordance. During the containment phase, patients with a positive blood ZIKV PCR test were admitted to TTSH for clinical management and isolation to reduce the risk of community transmission, while those who only had a positive urine ZIKV PCR test were discharged. All inpatients underwent daily blood ZIKV PCR testing and were discharged only when their blood tested negative for ZIKV.

Our study evaluated patients who had a positive blood sample for ZIKV by rRT-PCR testing, and correlated their period of viremia with their clinical and laboratory characteristics. Within this group of patients, we excluded those with discordant blood ZIKV PCR results between the two testing laboratories, those who had concurrent bacterial or viral infections on admission, and those who did not have a documented negative blood ZIKV PCR prior to discharge. Only blood samples that tested positive from both laboratories were included in the analysis to improve diagnostic accuracy as the testing platform was relatively new. Serial testing of urine for ZIKV PCR was not performed as it was used only at the point of presentation to confirm the diagnosis.

Clinical data, daily symptoms and signs and laboratory results for all patients were obtained from electronic medical records. A standardised ZIKV disease clinical care pathway was used for recording daily signs and symptoms, vital signs and management of all hospitalised patients. Clinical data collected included demographic information, presence of medical co-morbidities, travel history and contact history. Baseline investigations for all confirmed cases of ZIKV disease included a full blood count, renal function, liver function and dengue serology. Further investigations such as blood cultures and chest X-ray were performed if clinically indicated. The reference values for the normal range of laboratory tests were in accordance with those used by the hospital’s laboratory. Duration of viremia was calculated from the day of onset of symptoms until the first negative blood ZIKV PCR result. Duration of clinical illness was calculated from the first day of onset of symptoms to the day of resolution of all symptoms.

Key laboratory parameters were visualised with box plots to investigate the overall trend over the course of ZIKV infection. Chi-square and Fisher’s exact tests were used to evaluate differences in proportions for categorical variables, while Mann-Whitney U test was used to evaluate differences in medians for continuous variables. A *p* value of < 0.05 was taken to be statistically significant. All statistical analyses were performed using Stata version 13 (StataCorp 2013, College Station, TX).

## Results

**Fig. 1:**
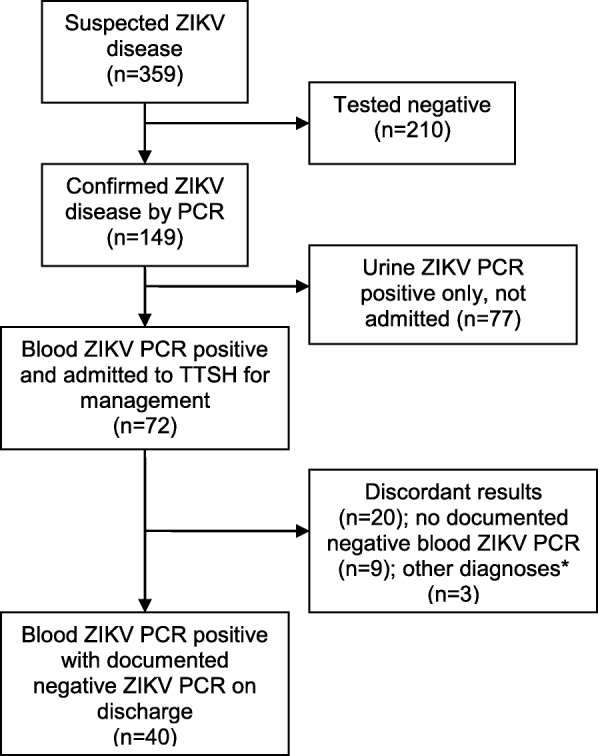
Flowsheet of patients with suspected and confirmed ZIKV disease. * *One patient with dengue co-infection, one with cellulitis and one with mediastinal mass for investigation*

A total of 359 patients were referred to the CDC for suspected ZIKV disease from 26 August to 5 September 2016. (Fig. 1) Of these, 72 patients yielded a positive blood ZIKV PCR test at the time of presentation, and were admitted to the CDC for isolation. Twenty patients were excluded from the analysis due to differences in blood results between the two laboratories. Another nine were excluded as they had no available negative blood ZIKV PCR result to indicate the end of viremia: five were discharged at the end of the containment phase with no negative blood PCR result available, and four patients had been transferred to another hospital. Of the remaining 43 patients, a further three were excluded as one had dengue co-infection, one had cellulitis, while the remaining patient had been admitted for investigation of a mediastinal mass and was incidentally found to have ZIKV infection.

Of the 40 inpatients, 24 (60%) were male and 31 (76%) were of Chinese ethnicity. Thirty-four (85%) were Singaporeans while the remainder were foreigners residing in Singapore. The median age was 34 years [inter-quartile range (IQR) 26 to 52.5 years]. Five of them reported travel in the last 14 days prior to admission, all to Malaysia. None of the female patients was pregnant.

### Clinical presentation and correlation with viremia

Among the 40 viremic patients, the median duration of viremia was 3.5 days (IQR: 3 to 5 days), while the median duration of symptoms was 3 days (IQR: 2 to 4 days). Table 1 documents the daily progression of combined signs and symptoms from onset of illness to the end of viremia.

**Table 1:**
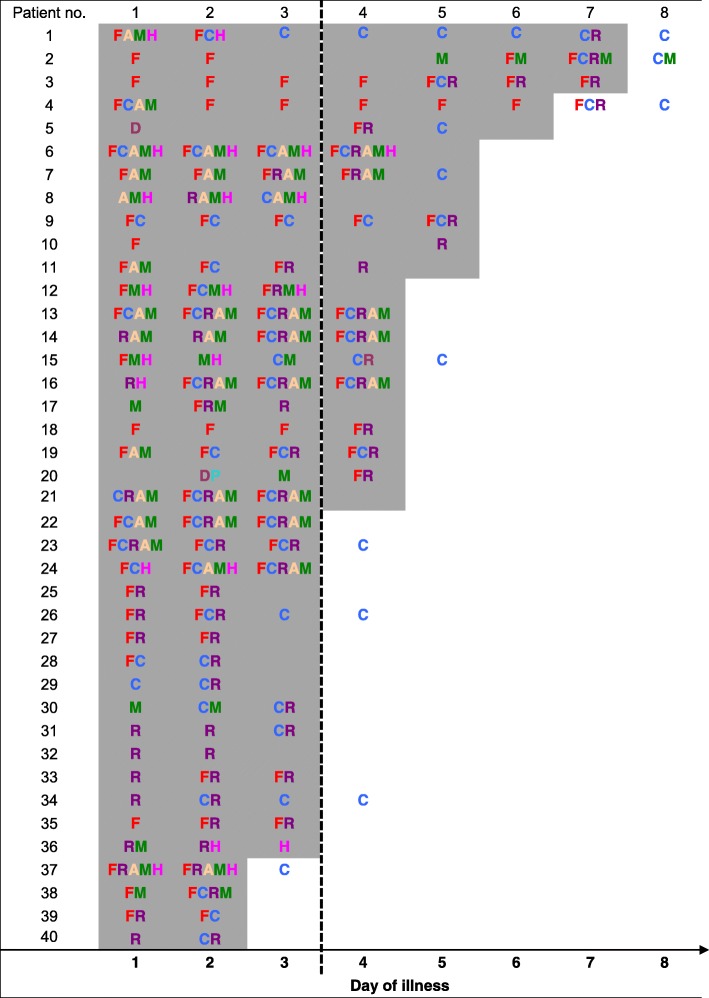
Daily trend of symptoms and signs with viremia in 40 patients with ZIKV disease

Positive ZIKV PCR.

Negative ZIKV PCR.

*F* fever, *C* conjunctivitis, *R* rash, *H* headache, *A* arthralgia, *M* myalgia, *D* diarrhea, *P* abdominal pain.

Of the signs and symptoms, rash was the most common, being present in all patients. (Table 2) The rash was noted to be generalised and maculopapular in 31 of 40 patients (79%) but distribution and character were unknown in 9 patients. The next most common sign and symptom was fever, present in 32 patients (80%); followed by myalgia in 25 patients (62.5%), conjunctivitis in 24 patients (60%) and arthralgia in 15 patients (38%).

**Table 2:** Correlation of symptoms, signs and laboratory investigations on admission with viremia

Symptoms and signs on admission	Viremia < 4 days (*n* = 19) *n*, (%)	Viremia ≥4 days (*n* = 21) *n*, (%)	*p*-value
Fever^a^	12 (63%)	20 (95%)	0.01
Conjunctivitis^a^	10 (53%)	14 (67%)	NS
Rash^a^	19 (100%)	21 (100%)	NS
Arthralgia	5 (26%)	10 (48%)	NS
Myalgia	9 (47%)	16 (76%)	NS
Headache	4 (21%)	6 (29%)	NS
Diarrhea	3 (16%)	3 (14%)	NS
Abdominal pain	0 (0%)	1 (5%)	NS
Nausea/ vomiting	0 (0%)	1 (5%)	NS
Disorientation	0 (0%)	0 (0%)	NS
Jaundice	0 (0%)	0 (0%)	NS
Lymphadenopathy	1 (5%)	1 (5%)	NS
Pharyngitis	1 (5%)	3 (14%)	NS
Abnormal lung findings: crackles/ wheeze	0 (0%)	0 (0%)	NS
Laboratory investigations (median, IQR)			
White cell count, 10^9^/L	3.6 (3–10.3)	5.3 (2.7–6.5)	NS
Hemoglobin, g/dL	13.6 (10.5–17.9)	14.8 (12.6–16.4)	NS
Platelet count, 10^9^/L	176 (117–339)	188 (99–291)	NS
Hematocrit, %	41.5 (33.9–52.5)	45 (36–51.1)	NS
Potassium, mmol/L	3.45 (3.2–3.8)	3.65 (2.3–3.9)	NS
Urea, mmol/L	3.4 (2.4–4.9)	2.1 (1.9–3.4)	NS
Creatinine, unit	68 (47–95)	60 (46–112)	NS
Neutrophils	2.17 (1.73–4.67)	3.21 (1.06–4.66)	NS
Lymphocytes	1.14 (0.63–4.28)	1.12 (0.58–1.72)	NS
AST, IU	26 (18–40)	30 (17–124)	NS
ALT, IU	20 (14–71)	22 (15–196)	NS
Bilirubin	12 (8–13)	11 (9–19)	NS
Albumin, g/L	39 (37–46)	39 (32–43)	NS
Number of initial symptoms and signs	2 (1–2)	3 (1–3)	NS

^a^denotes combined signs and symptoms

The duration of symptoms did not exceed the duration of viremia in 32 (80%) patients. Eight patients had symptoms beyond the duration of viremia, for which conjunctivitis was the predominant persisting symptom. Of these 8 patients, fever exceeded the duration of viremia only in one patient (subject 4). Of the 32 patients who had reported or documented fever, the end of viremia corresponded with defervescence in 17 patients (53%). However, in 12 patients (30%), viremia persisted for 1 day beyond the resolution of symptoms.

Vital signs were all within normal parameters, with no patients having tachypnea (respiratory rate > 28 breaths per minute), tachycardia (pulse rate > 100 beats per minute) or hypotension (systolic blood pressure < 90 mmHg). None of the patients had postural hypotension. All patients remained well during the course of illness with good recovery and none developed bleeding or neurological symptoms. There were no admissions to the intensive care unit and none of the patients was readmitted in the 3 months following their illness.

We divided the cohort into two groups based on duration of viremia, using a cut-off of 4 or more days to denote prolonged viremia. A higher proportion of patients with reported fever on the first day of illness was observed among patients with prolonged viremia (95%), compared to those without prolonged viremia (63%). There were otherwise no significant differences in symptoms and signs between the two groups. (Table 2) There was also no association between duration of viremia and the number of initial symptoms and signs (*p* = 0.26).

Laboratory results generally remained normal in this cohort, regardless of the duration of viremia. Leukopenia was not common, with only 17 patients (24%) having a white cell count < 4.0 × 10^9^/L and the lowest being 2.6 × 10^9^/L. Only 3 patients had thrombocytopenia (platelet count < 100 × 10^9^/L), the lowest being 83 × 10^9^/L. None of the patients showed significant hemoconcentration. Interestingly, 21 patients had hypokalemia (potassium level < 3.5 mmol/L) despite the absence of anorexia or gastrointestinal symptoms. Of these, five patients had moderate hypokalemia (3.0–3.5 mmol/L), while one patient had severe hypokalemia (2.3 mmol/L). Only 5 patients had a creatinine of more than 105 umol/L, but none of the readings were more than 1.5 times their baseline (if known). Two of them had mild hypokalemia. Only one patient had an elevated aspartate aminotransferase (AST) of 174 U/L and alanine aminotransferase (ALT) of 253 U/L but further workup showed that the patient subsequently tested positive for Hepatitis C. Analysis of the laboratory investigations between the two groups also showed that there were no significant differences in values.

**Fig. 2:**
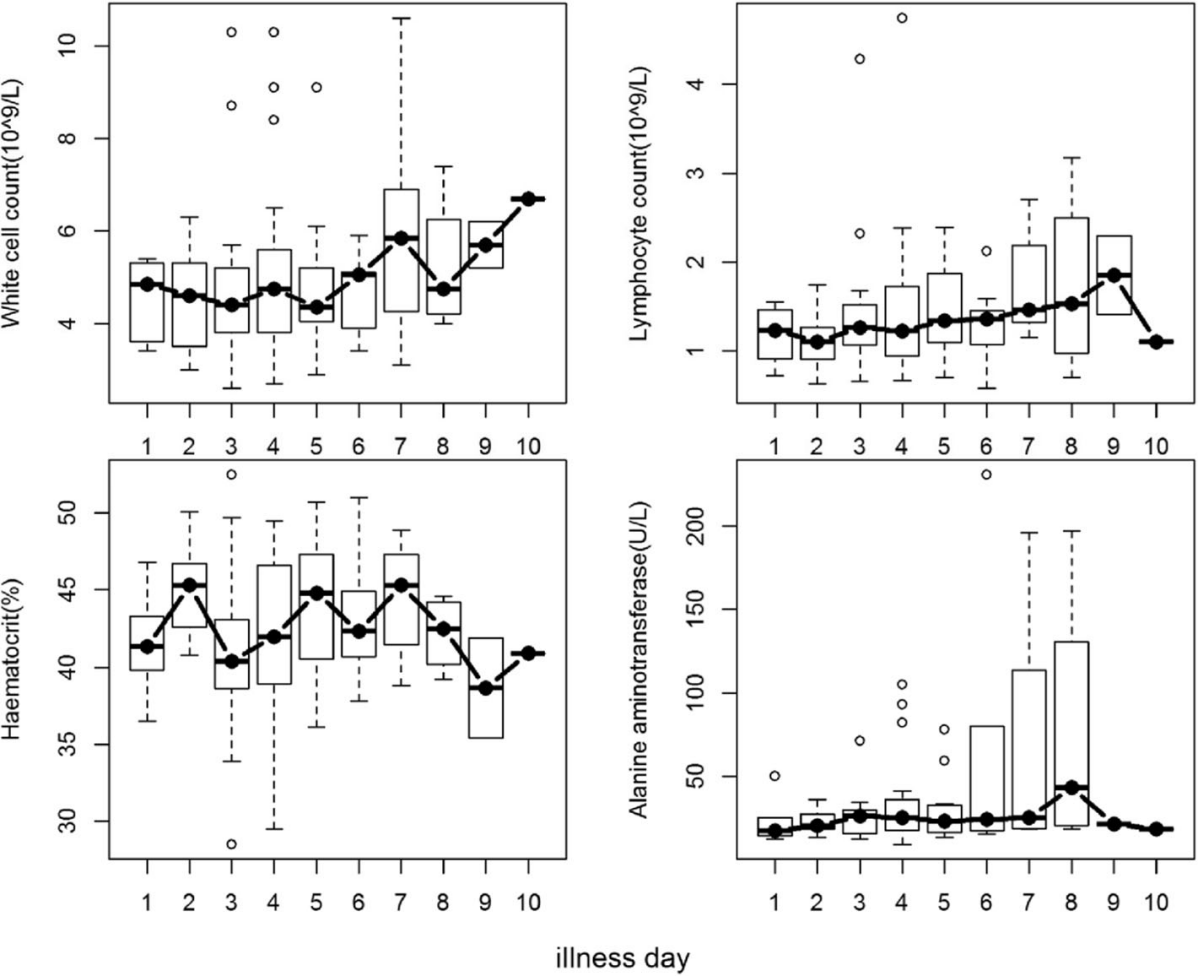
Box plots of laboratory markers by day of illness

Box plots of the white cell count, lymphocyte count, hematocrit and ALT plotted by day of illness also showed that there were no significant differences in laboratory values for each day of illness. (Fig. 2).

## Discussion

In our cohort of blood ZIKV PCR positive patients, clinical presentation was characterised by symptoms and signs of rash, fever, myalgia, arthralgia and non-purulent conjunctivitis, with only a small proportion reporting gastrointestinal symptoms such as diarrhea, abdominal pain, nausea and/or vomiting. This was similar to a study of 57 patients with ZIKV disease in Brazil, which also reported rash as the most common symptom, followed by headache, fever, arthralgia and myalgia [7].

We observed that 63% of patients reported or were documented to have fever on the first day of illness, suggesting that this could be the start of viremia (Table 1). In addition, fever showed better correlation with the period of viremia than other symptoms such as rash, myalgia and conjunctivitis. Nevertheless, a sizeable proportion of patients had persistent viremia even after fever had resolved. Other symptoms such as rash (the most commonly reported symptom) and conjunctivitis tended to appear only toward the middle or end of the illness. In addition, over a third of patients were no longer symptomatic while remaining viremic. Our findings differ from that of dengue, another flavivirus, where viremia has been reported to correlate with defervescence [8], and suggest that the neither the duration of any symptoms of ZIKV, nor the total number of symptoms on presentation, correlate well with the duration of viremia. While patients are often counselled to practise safe sex, owing to reports of sexual transmission of ZIKV [9], our findings suggest the possibility of continued transmission to the *Aedes* mosquito vector even after resolution of symptoms and signs. Hence, they support the continued use of personal protective measures to prevent mosquito bites and mosquito control interventions and the continuation of environmental public health measures to reduce the mosquito population to reduce transmission risk.

Previous studies have reported laboratory abnormalities such as leukopenia, neutropenia, thrombocytopenia or deranged liver function tests [10]. However, findings of our study demonstrated that these abnormalities are generally uncommon, with none of the patients with ZIKV disease having abnormal liver function tests. In addition, there were no significant variations in laboratory values across each day of illness. Interestingly, however, half our patients were found to have hypokalemia despite the absence of anorexia or symptoms to suggest gastrointestinal loss. Dengue fever has been reported to cause proteinuria or glomerulonephritis [11]. Similarly, the presence of ZIKV in the urine suggests that there is a possibility of the virus also causing glomerular injury, resulting in renal potassium loss. An in vitro study of renal cells infected with ZIKV showed that there were high levels of viral replication within different renal cell types, especially podocytes, the damage to which could initiate progressive renal impairment [12]. In our cohort, none of the patients had evidence of renal impairment. However, we were unable to conduct detailed studies of renal function or trend the results. Further studies are required to correlate the level of viruria with the degree of renal impairment and hypokalaemia. Nonetheless, from a clinical perspective, it may be prudent to also check potassium levels in patients diagnosed with ZIKV disease.

A strength of this paper is the well-characterised Asian cohort, with daily symptoms and signs clearly documented according to a standardised clinical care pathway. Limitations of the study include the small size of the cohort, and the subjectivity of symptoms and possible under-reporting by patients. It was therefore difficult to ascertain if the duration and level of viremia had any impact on development of future complications, such as GBS.

## Conclusions

This is the first known paper examining the correlation of symptoms and signs of viremia in ZIKV disease in an Asian cohort. Our study demonstrated that the duration of ZIKV viremia did not correlate well with the progression of clinical disease or symptoms, unlike that seen in that of dengue fever. In addition, a sizeable proportion of patients may still be viremic despite resolution of symptoms and signs. While laboratory abnormalities were not a prominent feature of ZIKV disease, over half the patients had hypokalemia, which may be suggestive of direct glomerular injury by the virus, although further pathological studies are required to confirm this.

## Abbreviations

ALT: alanine aminotransferase
AST: aspartate aminotransferase
CDC: Communicable Disease Center
GBS: Guillain-Barré syndrome
MOH: Ministry of Health
PCR: polymerase chain reaction
RNA: ribonucleic acid
rRT-PCR: Real-time reverse transcriptase polymerase chain reaction
TTSH: Tan Tock Seng Hospital
WHO: World Health Organisation
ZIKV: Zika virus
